# Practical and Emotional Problems Reported by Users of a Self-guided Digital Problem-solving Intervention During the COVID-19 Pandemic: Content Analysis

**DOI:** 10.2196/31722

**Published:** 2021-10-04

**Authors:** Amira Hentati, Erik Forsell, Brjánn Ljótsson, Martin Kraepelien

**Affiliations:** 1 Centre for Psychiatry Research Department of Clinical Neuroscience Karolinska Institutet & Stockholm Health Care Services, Region Stockholm Stockholm Sweden; 2 Division of Psychology Department of Clinical Neuroscience Karolinska Institutet Stockholm Sweden

**Keywords:** digital intervention, COVID-19, problem-solving, self-guided intervention, content analysis, public health, mental health, depression, anxiety, pandemic

## Abstract

**Background:**

To better direct assessments and interventions toward the general population during both the ongoing COVID-19 pandemic and future crises with societal restrictions, data on the types of practical and emotional problems that people are experiencing are needed.

**Objective:**

The aim of this study was to examine the types of practical and emotional problems that the general population is experiencing during the COVID-19 pandemic and to construct an empirically derived inventory based on the findings.

**Methods:**

A total of 396 participants, recruited among members of the general public in Sweden who were experiencing practical and/or emotional problems during the pandemic, accessed a self-guided digital problem-solving intervention for a period of 1 week to report and solve the problems they experienced. Prior to accessing the intervention, the participants completed a short self-assessment regarding symptoms of depression and anxiety. Content analysis was used to account for the types of problems participants reported. A set of items for an inventory was later proposed based on the problem categories derived from the analysis.

**Results:**

A majority of participants had clinically relevant symptoms of either depression or anxiety. The problems reported were categorized as 13 distinct types of problems. The most common problem was difficulty managing daily activities. Based on the categories, a 13-item inventory was proposed.

**Conclusions:**

The 13 types of problems, and the proposed inventory, could be valuable when composing assessments and interventions for the general population during the ongoing pandemic or similar crises with societal restrictions. The most common problem was of a practical nature, indicating the importance of including examples of such problems within assessments and interventions.

**Trial Registration:**

ClinicalTrials.gov NCT04677270; https://clinicaltrials.gov/ct2/show/NCT04677270

## Introduction

### Background

The COVID-19 pandemic is considered a threat to the mental well-being of the general public and may increase the suicide risk for some people [1,2]. This threat consists of both emotional problems, such as anxiety, loneliness, and low mood [2,3], and practical problems, such as not being able to work remotely or to travel as before the pandemic [4,5].

Population-level efforts aiming to prevent negative mental health consequences have been called for since the first months of the COVID-19 pandemic [1]. Furthermore, remotely implementable digital interventions for treatment and prevention have been seen as critical to achieve the scalability necessary to have an impact on the health of the general public [6]. An example of a successful population-level effort during the COVID-19 pandemic is described in a previous study in which an existing intervention for extensive worry was adapted to COVID-19–related worry, transformed into a self-guided format [7], and later implemented in the Swedish regular health care system. However, because worry is only one possible problem experienced by the general population during the COVID-19 pandemic [8], there is a need to assess what types of practical and emotional problems the general population is experiencing. This could facilitate the direction of assessments and interventions toward the general public both during the ongoing pandemic and in future similar crises.

Problem-solving therapy is a well-examined intervention that was originally constructed for major depression, targeting the ability to solve problems [9]. Moreover, the ability to solve problems has been highlighted as one of several protective factors for individuals in the general population affected by the COVID-19 pandemic when considering societal suicide prevention [10]. A problem-solving intervention could thus be a suitable intervention for the general public experiencing practical or emotional problems during the ongoing pandemic. Ideally, it should be easy to gain access to such an intervention, and the intervention should be self-guided to facilitate scalability. An open access and self-guided internet-based psychological support intervention (PATH), which includes problem-solving as well as conflict management and stress management, has been examined with regard to participants’ input to the program during the COVID-19 pandemic [11]. In that study, conflicts with others, worry, and difficulties concentrating stood out as the most common types of problems during the pandemic.

### Aim

The aim of this study was to examine the types of practical and emotional problems that the Swedish help-seeking population is experiencing during the COVID-19 pandemic. An additional aim was to construct an empirically derived inventory to facilitate the assessment of problems and direction of interventions in the general population during the ongoing pandemic or similar crises with societal restrictions.

## Methods

### Setting and Study Design

This study was part of a project aiming to investigate treatment engagement with a self-guided digital problem-solving intervention between two different user interfaces. The results concerning the effect of the user interfaces on treatment engagement have been presented in a previous paper [12]. In this paper, the focus lies on the types of practical and emotional problems that the general help-seeking population in Sweden reported experiencing during the COVID-19 pandemic when using a self-guided digital problem-solving intervention. The study was approved by the Swedish national ethical review board (ID 2020-02739), and although the article does not report results of a health care intervention, the study was retrospectively registered on ClinicalTrials.gov (ID: NCT04677270, 2020-12-21).

### Participants and Recruitment

The target population was the general help-seeking population in Sweden who were experiencing practical and/or emotional problems during the COVID-19 pandemic. Participants were recruited nationwide in Sweden through advertisements on social media during a period of 6 weeks, between August 26 and October 6, 2020. Inclusion criteria were (1) age of 16 years or older, and (2) self-reported practical and/or emotional problems experienced during the COVID-19 pandemic. The second criterion was assessed through a question asking if participants experienced practical problems, emotional problems, practical and emotional problems, or no problems. No further definition of these problems was given at this stage of assessment.

### Procedures

**Figure 1:**
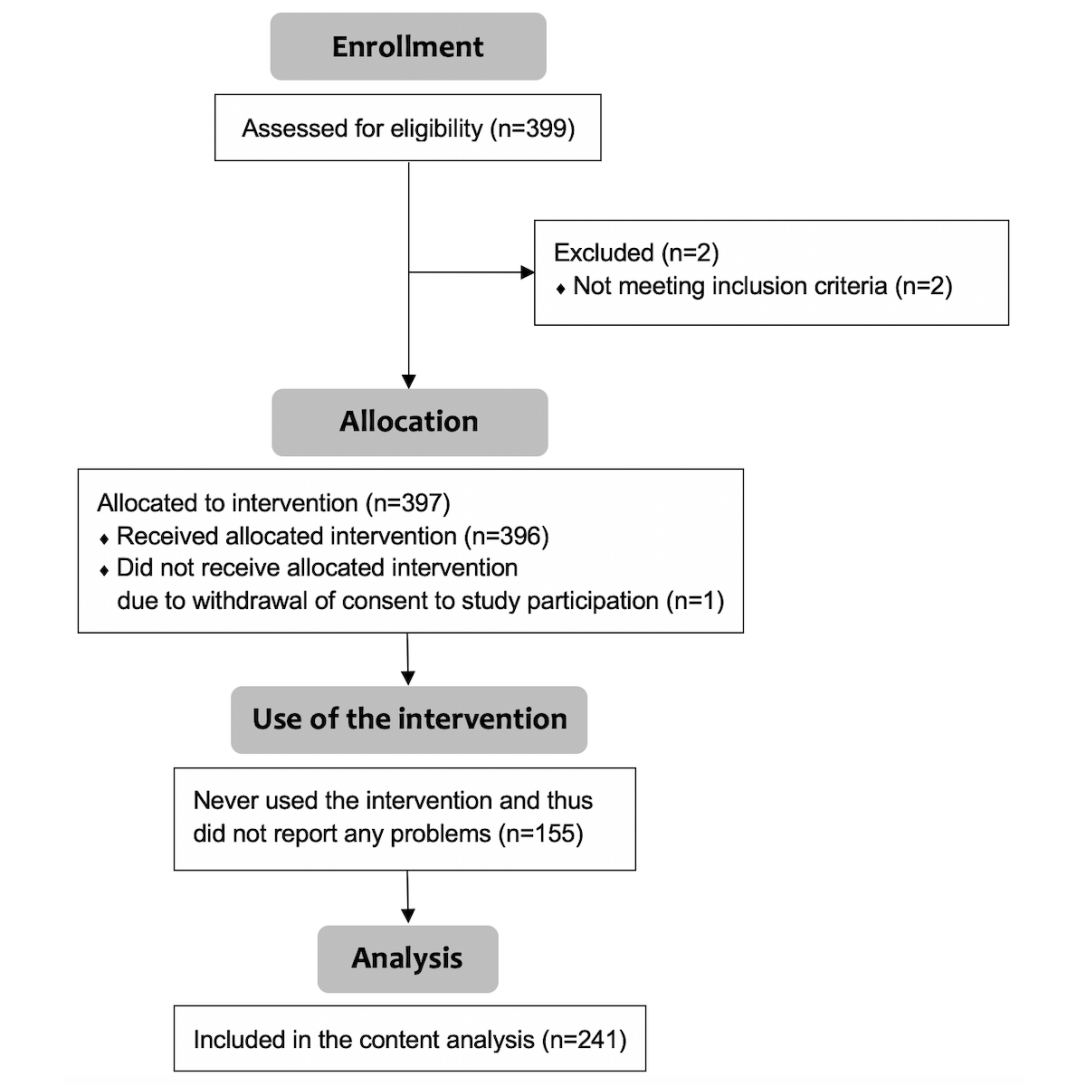
Flowchart of the selection of study participants.

To register for the study, participants completed a digital self-assessment on a secure digital platform. Written informed consent was digitally provided by all participants. Of the 399 individuals who registered for the study, only 2 persons were excluded from participating, owing to not experiencing problems (ie, the second inclusion criterion was not met). Furthermore, 1 person withdrew consent for participation. A total of 396 participants were thus included, and they accessed a self-guided digital problem-solving intervention for a period of one week to report and solve problems they experienced. The study flowchart is shown in Figure 1.

### Intervention

A self-guided digital problem-solving intervention adapted to the COVID-19 pandemic was created for the study. An already existing digital problem-solving intervention, previously used as a component in a 12-week multi-component internet-delivered cognitive behavioral therapy program for individuals with major depression in Swedish regular health care [13], was used as a template and adapted for self-guidance.

The problem-solving intervention comprised psychoeducational texts and rationale, examples of problems and suggestions of solutions, pictures, instructions, and problem-solving exercises. The exercises consisted of steps where participants were able to describe and choose to work with one problem at a time; set a goal; make a list of possible solutions to the problem, including pros and cons; choose what solutions to try and plan how and when to try them out; and then evaluate the solutions and reflect on possible lessons learned. All material within the intervention was in Swedish and was adapted to the pandemic situation. Participants could read texts about four fictive users having the following problems: health-related worry, stress because of their financial situation, loneliness, and problems with household activities. In total, the intervention consisted of a single module of approximately 4800 words. The length of the intervention was 1 week, because the current study only intended to examine types of problems and platform use (data presented in [12]) and not problem-solving as a clinical intervention. The intervention could be accessed on a secure digital platform via a computer or mobile device connected to the internet.

### Measurements

When registering for the study, participants completed a digital assessment comprising questions on demographics and whether they experienced problems during the COVID-19 pandemic. Participants also completed two self-assessed short scales, the Patient Health Questionnaire-2 (PHQ-2) [14] and Generalized Anxiety Disorder-2 (GAD-2) [15], measuring symptoms of depression and anxiety, respectively. These scales were administered to assess the proportion of participants with a possible clinical symptom burden, but they were not used either for inclusion or as an outcome measure.

### Data Analysis

Content analysis [16] was used to code all participants’ problem-solving attempts that were reported and saved within the digital platform as well as to create categories of problems based on these. All problem-solving attempts were entered in text on the digital treatment platform. Thus, transcription of data was not needed.

The step-by-step categorization of data started with defining each problem-solving attempt as the unit of analysis [17]. A problem-solving attempt was registered every time a participant completed the first step of the problem-solving exercises, which was to define the problem. Thus, a problem-solving attempt did not necessarily constitute a complete problem-solving exercise. To create categories for the content analysis, 25 participants were selected randomly for initial coding. Three of the authors, AH, EF, and MK, coded these participants jointly and created the coding instructions as well as the categories with consensus. Then, another 25 randomly selected participants were independently coded by the three coders, using the instructions and categories created. The interrater agreement between the coders was substantial [18] (Cohen κ=0.66-0.76). After checking that the interrater reliability was acceptable, disagreements were discussed until a consensus was reached. Afterward, the remaining 346 participants were divided between the three coders, and their problem-solving attempts were coded using the identified categories. Any uncertain categorizations were discussed with all coders. Each category was defined in turn as mainly either a practical or emotional problem.

The categorization of reported problem-solving attempts was later used in a quantitative description of the content [17]: namely, the number of problem-solving attempts that belonged to each category, as well as the number of the participants who had solved at least one problem that belonged to each category. Furthermore, to ensure that the data were not skewed by a few active participants, the use of the intervention was quantified by the percentage of participants using the intervention at least once, as well as the average number of problem-solving attempts completed during the week of access. Lastly, based on the problem categories derived from the analysis, a set of items for an inventory was proposed.

## Results

A majority of participants were women, were university educated, and had clinically relevant symptoms of either depression or anxiety. Table 1 shows the complete sample characteristics.

A majority of participants used the problem-solving intervention at least once during the week of access. Table 2 shows details on the use of the intervention.

The problems reported within the intervention by the participants who used the intervention at least once were categorized into 13 distinct types of problems, which can be found in Table 3. The 3 most frequent categories of problems were of a practical nature, such as difficulties initiating daily activities, problems or frustration regarding one’s work and/or study situation, and problems or frustration with public health guidelines or the pandemic situation in general.

Based on the 13 distinct types of problems derived from the content analysis (see Table 3), the items shown in Textbox 1 are proposed as an inventory of practical and emotional problems during the COVID-19 pandemic.

**Table 1:** Sample characteristics (N=396).

Variable		Value
Female gender, n (%)		352 (88.9)
**Age (years)**		
	Mean (SD)	40 (13)
	Range	17-79
In a relationship, n (%)		246 (62.1)
**Occupational status, n (%)**		
	Employed full-time	200 (50.5)
	Employed part-time	41 (10.4)
	Student	68 (17.2)
	Parental leave	7 (1.8)
	Unemployed	34 (8.6)
	Long-term sick leave	21 (5.3)
	Retired	25 (6.3)
**Education, n (%)**		
	Primary school	7 (1.8)
	Secondary school	78 (19.7)
	University	311 (78.6)
Possible major depression (PHQ-2^a^ score ≥3), n (%)		235 (59.3)
Possible generalized anxiety (GAD-2^b^ score ≥3), n (%)		236 (59.6)
Concurrent possible depression and anxiety, n (%)		180 (44.5)
Either possible depression, anxiety, or both, n (%)		291 (73.5)

^a^PHQ-2: Patient Health Questionnaire-2.

^b^GAD-2: Generalized Anxiety Disorder-2.

**Table 2:** Use of the problem-solving intervention (N=396).

Variable	Value
Used the problem-solving intervention at least once, n (%)	241 (60.9)
Problem-solving attempts per participant, mean (SD)	1.13 (1.44)

**Table 3:** Types of problems identified from the content analysis.

Problem type	Problem category	Number of participants with problem (% of number of participants who used the intervention at least once, N=241)	Number of problems (% of total number of reported problems, N=446)	Definition	Examples
Daily activities	Practical	51 (21.2)	59 (13.2)	Difficulties initiating daily activities, staying motivated, or maintaining focus	Not getting household activities done Spending too much time on social media
Work and study	Practical	46 (19.1)	51 (11.4)	Problems or frustration regarding work and/or study situation	Working at home with children Struggling with digital work or studies
Health behaviors	Practical	40 (16.6)	44 (9.9)	Difficulties maintaining health promoting behaviors such as physical activity, satisfactory sleep patterns, or active recovery	Getting less physical exercise than usual Having trouble falling asleep
Family and relationship	Emotional	37 (15.4)	40 (9)	Problems related to relationships, including family, friends, or significant other	Feeling unhappy in a relationship Finding it difficult to establish new relationships
Health anxiety	Emotional	35 (14.5)	36 (8.1)	Affected emotionally by health fears or worry regarding self or others	Worrying about being infected with COVID-19 Worrying that relatives or friends will become ill with COVID-19
Pandemic guidelines	Practical	31 (12.9)	45 (10.1)	Problems or frustration with public health guidelines or the pandemic situation in general	Being bound to home as soon as you experience the sightliest signs of symptoms of COVID-19 Feeling frustrated at others not following the pandemic guidelines
Non–health-related anxiety or stress	Emotional	30 (12.4)	35 (7.8)	Affected emotionally by non–health-related anxiety or stress-related problems	Experiencing social anxiety or generalized anxiety Feeling overwhelmed
Financial issues	Practical	26 (10.8)	30 (6.7)	Financial problems or fears	Experiencing fear of losing your job Experiencing loss of income
Loneliness	Emotional	22 (9.1)	22 (4.9)	Affected emotionally by loneliness	Negatively affected by having had less contact with family or friends Experiencing social isolation
Low mood	Emotional	20 (8.3)	21 (4.7)	Low mood or feelings of meaninglessness	Feeling sad most of the time Experiencing apathy
Changes in emotional state apart from anxiety and low mood	Emotional	19 (7.9)	22 (4.9)	Emotional challenges other than anxiety or low mood	Feeling angry Experiencing low self-esteem
Health issues	Practical	19 (7.9)	23 (5.2)	Health issues related to COVID-19 or other illness regarding self or others	Experiencing difficult symptoms of COVID-19 or other illness Having a relative or friend with health issues
Weight and eating	Practical	18 (7.5)	18 (4)	Problems related to weight or eating	Experiencing unintentional weight change Struggling with binge eating

**Proposed inventory of practical and emotional problems during a crisis with societal restrictions.**

**Practical problems: do you experience…**

- Difficulties initiating daily activities, staying motivated or maintaining focus? Examples of these difficulties include not getting household activities done, or spending too much time on social media.
- Problems or frustration regarding your work and/or study situation? Examples of these problems include working at home with children, or struggling with digital work or studies.
- Difficulties maintaining health promoting behaviors such as physical activity, satisfactory sleep patterns, or active recovery? Examples of these difficulties include getting less physical exercise than usual, or having trouble falling asleep.
- Problems or frustration with public health guidelines or the pandemic situation in general? Examples of these problems include being bound to home as soon as you experience the sightliest signs of symptoms of COVID-19, or feeling frustrated at others not following the pandemic guidelines.
- Financial problems or fears? Examples of these problems include experiencing fear of losing your job or of losing income.
- Health issues related to COVID-19 or other illness regarding self or others? Examples of these problems include experiencing difficult symptoms of COVID-19 or other illness, or having a relative or friend with health issues.
- Problems related to weight or eating? Examples of these problems include experiencing unintentional weight change, or struggling with binge eating.

**Emotional problems: do you experience…**

- Problems related to relationships, including with your family, friends, or significant other? Examples of these problems include feeling unhappy in a relationship, or finding it difficult to establish new relationships.
- Being affected emotionally by health fears or worry regarding yourself or others? Examples of these difficulties include fearing or worrying about being infected with COVID-19, or worrying that relatives or friends will become ill with COVID-19.
- Being affected emotionally by non–health-related anxiety or stress? Examples of these difficulties include experiencing social anxiety or generalized anxiety, or feeling overwhelmed.
- Being affected emotionally by loneliness? Examples of these difficulties include being negatively affected by having had less contact with family or friends, or experiencing social isolation.
- A low mood or feelings of meaninglessness? Examples of these problems include feeling sad most of the time, or experiencing apathy.
- Emotional challenges other than anxiety or low mood? Examples of these difficulties include feeling angry, or experiencing low self-esteem.

## Discussion

### Principal Results

In this study, COVID-19–related practical and emotional problems experienced by the Swedish help-seeking population were examined during the use of a self-guided digital problem-solving intervention. Content analysis was used to investigate the types of problems reported within the intervention.

The participants reported 13 different distinct types of problems. Practical problems, such as managing one’s daily life and work situation, were most frequently reported, while loneliness, low mood, and other emotional difficulties were less common. The dominance of practical problems may have been due to participants preferring to use the digital problem-solving intervention to generate solutions to practical problems rather than emotional problems. However, it may also have to do with practical problems actually being the predominant problem type experienced by participants in this study. Additionally, it has been reported that the COVID-19 pandemic has had a vital impact on people’s practical work situations [19]; societal restrictions have also led to great challenges in managing work-family balance, with sometimes minor support [20,21]. It should be further noted that the short scales PHQ-2 and GAD-2, which were used to assess the proportions of participants with possible major depression and anxiety, respectively, are probably quite sensitive to symptoms when used at the stage of screening [14]. This could help explain the high prevalence of possible clinical symptom burden among the participants, despite the fact that practical problems were more commonly reported than emotional ones.

In a previous study, COVID-19–related worry was highlighted as a target for a self-guided digital intervention [7]. Among the participants in the current study, health anxiety was reported, but it was not as common as problems of a more practical nature. This highlights the need to target a broad range of problems during a crisis involving a disease, including problems of a practical nature, as a complement to health-related worry.

The relatively low frequency of mood-related problems and feelings of loneliness may need to be interpreted within the context of the study being conducted in Sweden. Sweden has, unlike most other countries, not imposed mandatory lockdown during the COVID-19 pandemic. This may have impacted the mental well-being of the inhabitants. However, owing to voluntary restrictions recommended by the Swedish government during the pandemic, Sweden has had similar societal consequences, such as economic damage, to those of countries in lockdown [22]. As the results show, a number of people reported financial problems. These results can further be compared to the problems entered during the pandemic in the previously mentioned PATH program, which was based in the United States [12]. In that study, the common types of problems reported were similar to those in the current study, except for a greater emphasis on interpersonal conflicts, possibly due to the separate conflict management module in the PATH program. In most cases, these types of problems would fall into the category of problems related to relationships in the proposed inventory of the current study.

Although vaccination for COVID-19 has begun worldwide, it is still not clear whether some problems, both societal and health-related, that have arisen during the pandemic will persist for some time [23,24]. We believe that the inventory proposed in this paper of practical and emotional problems during a crisis with societal restrictions could therefore be of value not only during the still ongoing pandemic but also possibly in the near future. We propose that the inventory can act as a guide when constructing both assessments and interventions related to COVID-19 problems while also providing some public health information concerning the pandemic and its consequences.

Because most items in the proposed inventory are not specific to COVID-19, the inventory may also provide helpful guidance in future similar crises. However, it is not certain that all the items will be relevant in a future crisis with societal restrictions. Health anxiety, which was reported in this study, is an example of a type of problem that is more likely to occur during crises involving a disease. However, most other items derived from the content analysis fit into a crisis with societal restrictions whether or not a disease is involved.

Based on the fact that recruitment to the study was rapid, during a relatively short period of time, we interpreted the interest in gaining access to a problem-solving intervention as high. This reflects an apparent desire for interventions of this kind. We believe that this has practical implications, as the results from this study can be used to adapt and possibly improve similar interventions, both during the still-ongoing pandemic as well as for possible persisting problems that arose due to the pandemic.

When providing digital interventions in a self-guided format, there is a risk of low adherence and low use. This was exemplified in a previous Swedish adaptation of a self-guided intervention for mental health problems [25]. We believe that one of the strengths of the current study is that a majority of participants used the digital intervention and hence contributed to the generalizability of the results.

### Limitations

There are some limitations to this study that need to be acknowledged. First, the sample predominantly consisted of university-educated women, which impacts the generalizability of the results. Second, participants were recruited through advertisements on social media. We are not sure if the results or sample would have differed if additional recruitment methods would have been used. Third, we have no available data concerning problems reported by the current population before the COVID-19 pandemic, making it difficult to discern the impact of the pandemic on the problems reported. Fourth and last, the problem-solving intervention constructed for individuals with major depression that was used as a template for the development of the digital intervention used in the current study is intended to be used over several weeks [13]. In the current study, access to the intervention was limited to a period of 1 week. It is unclear whether a longer period of access to the intervention would have resulted in participants reporting additional types of problems.

### Future Research

For future studies, we recommend that the inventory suggested within this paper be evaluated with regard to psychometric properties.

### Conclusions

The reported problems of participants during the COVID-19 pandemic in this study fell into 13 distinct categories of problems. These can serve as targets of interventions or be of help when screening for problems in the general population during the ongoing pandemic or in future similar crises. The most frequently reported types of problems were of a practical nature, indicating the importance of giving examples of practical problems within both interventions and assessments.

## Abbreviations

GAD-2: Generalized Anxiety Disorder-2
PHQ-2: Patient Health Questionnaire-2
